# Rad51 Interacts with Non-structural 3 Protein of Hepatitis C Virus and Regulates Viral Production

**DOI:** 10.3389/fmicb.2017.01249

**Published:** 2017-07-06

**Authors:** Kidong Son, Tram T. T. Nguyen, Jae-Woong Choi, Long V. Pham, Trang T. D. Luong, Yun-Sook Lim, Soon B. Hwang

**Affiliations:** ^1^Department of Biomedical Gerontology, Graduate School of Hallym UniversityChuncheon, South Korea; ^2^National Research Laboratory of Hepatitis C Virus and Ilsong Institute of Life Science, Hallym UniversityAnyang, South Korea; ^3^Environmental Health Research Department, National Institute of Environmental ResearchIncheon, South Korea

**Keywords:** hepatitis C virus, Rad51, siRNA screening, NS3, therapeutic target, viral production

## Abstract

Hepatitis C virus (HCV) is a leading cause of chronic liver disease affecting over 170 million people worldwide. Chronic infection with HCV progresses to liver fibrosis, cirrhosis, and hepatocellular carcinoma. HCV exploits host cellular factors for viral propagation. To investigate the cellular factors required for HCV propagation, we screened a siRNA library targeting human cell cycle genes using cell culture grown HCV-infected cells. In the present study, we selected and characterized a gene encoding Rad51. Rad51, a member of a conserved recombinase family, is an essential factor for homologous recombination and repair of double-strand DNA breaks. We demonstrated that siRNA-mediated knockdown of Rad51 significantly inhibited HCV propagation without affecting HCV RNA replication. Silencing of Rad51 impaired secretion of infectious HCV particles and thus intracellular viruses were accumulated. We showed that HCV NS3 specifically interacted with Rad51 and accumulated Rad51 in the cytosol. Furthermore, Rad51 was coprecipitated with NS3 and HCV RNA. By employing membrane flotation and protease protection assays, we also demonstrated that Rad51 was co-fractionated with HCV NS3 on the lipid raft. These data indicate that Rad51 may be a component of the HCV RNA replication complex. Collectively, these data suggest that HCV may exploit cellular Rad51 to promote viral propagation and thus Rad51 may be a potential therapeutic target for HCV.

## Introduction

Hepatitis C virus is a major causative agent of chronic hepatitis, cirrhosis, and HCC (Saito et al., 1990). Approximately 150–170 million people worldwide are chronically infected with HCV. HCV is an enveloped virus with a positive-sense, single-stranded RNA genome. HCV belongs to the genus *Hepacivirus* in the family *Flaviviridae*. The HCV genome encodes a single precursor polyprotein, which is cleaved by both cellular and viral proteases to generate three structural (core, E1, and E2) and seven non-structural (p7; NS2 to NS5B) proteins (Lindenbach and Rice, 2005). Non-structural 3 (NS3) is a multifunctional protein which displays serine protease and RNA helicase activities. Both protease and helicase activities of NS3 are essential for viral protein processing and HCV replication (Welbourn et al., 2005). The NS3/4A protease has been shown to regulate cellular innate antiviral response by targeting MAVS for cleavage (Li K. et al., 2005; Li X.D. et al., 2005; Meylan et al., 2005).

Recently, numerous direct-acting antivirals (DAAs) have been developed to treat HCV patients with high cure rates. Despite the advent of effective DAAs, drug resistance-associated HCV variants occur due to low genetic barriers. Moreover, sustained virologic responses of DAAs vary depending on HCV genotypes (Kiser et al., 2013). As an alternative approach to develop potent antivirals, host-targeting antiviral (HTAs) may have some advantages in terms of high barrier to resistance and a potential for pan-genotypic antiviral activity (Pawlotsky, 2014).

Rad51 is an essential factor for homologous recombination and repair of DNA double-strand breaks. Rad51 binds transiently to both single-strand and double-strand DNA during the recombination reaction (Baumann and West, 1998). Homologous recombination is used for repair of DNA damage by binding of Rad51 to single-strand DNA to form the Rad51 nucleofilament (Sung, 1997). Recently, it has been reported that the human Rad51 protein interacts with HIV-1 integrase and inhibits its activity (Cosnefroy et al., 2012; Thierry et al., 2015). Moreover, Rad51 modulates hepatitis B virus (HBV) infection by maintaining genome integrity and mediating homologous DNA repair (Pasaje et al., 2011). In the present study, we showed that silencing of Rad51 impaired secretion of infectious HCV particles without affecting HCV replication. We also demonstrated that Rad51 interacted with both NS3 and HCV RNA. Moreover, Rad51 was co-fractionated with HCV NS3 on the lipid raft, suggesting that it may be a component of the HCV RNA replication complex. These data suggest that HCV may coopt host Rad51 for its own propagation.

## Materials and Methods

### Cell Culture

All cell lines were grown in Dulbecco’s modified Eagle’s medium (DMEM) supplemented with 10% fetal bovine serum and 100 units/ml penicillin/streptomycin in 5% CO_2_ at 37°C. Both Huh7 cells harboring subgenomic replicon derived from genotype 1b and Huh6 cells harboring subgenomic replicon derived from genotype 2a were grown as reported previously (Lim et al., 2011).

### Plasmid Constructions and DNA Transfection

Plasmids expressing Myc-tagged NS3 (genotype 1b), NS5A, and NS5B have been described previously (Lim et al., 2011). NS3/4A derived from genotype 2a was amplified by PCR and constructed in pEF6/Myc vector (Invitrogen). Full-length RAD51 was amplified by PCR from cDNA synthesized using total RNA of Huh7.5 cells and constructed into plasmid p3XFLAG-CMV10 (Sigma–Aldrich) and pEF6/V5 (Invitrogen). For the transfection experiment, cells were transfected with the indicated plasmids using polyethyleneimine reagent (Sigma) as we reported previously (Lim et al., 2011).

### Antibodies

Antibodies were purchased from the following source: RAD51 (sc-8349), Myc (sc-40), and GAPDH (sc-25778) antibodies were from Santa Cruz, β-actin (5441) and Flag antibodies (F7425) were from Sigma–Aldrich, and V5 antibody was from Invitrogen. HCV core, NS3, NS5A antibodies were described elsewhere (Lim et al., 2011).

### RNA Interference

siRNAs targeting Rad51 (#1, 5′-UAU CAU CGC CCA UGC AUC A-3′; #2, 5′-CUA AUC AGG UGG UAG CUC A-3′; #3, 5′-CCA ACG AUG UGA AGA AAU U-3′; #4, 5′-GCA GUG AUG UCC UGG AUA A-3′) were purchased from Dharmacon (Lafayette, CO). The universal negative control siRNA (SN-1003) was purchased from Bioneer (Korea). siRNA targeting 5′NTR of Jc1 (5′-CCU CAA AGA AAA ACC AAA CUU-3′) was used as a positive control (Randall et al., 2007). The siRNA targeting sequences for SPCS1 and ApoE were 5′-CUC UCA AGU GGU UAC CUG U-3′ and 5′- AGA CAG AGC CGG AGC CCG A-3′. siRNA transfection was performed using a Lipofectamine RNAiMax reagent (Invitrogen, Carlsbad, CA, United States) according to the manufacturer’s instructions.

### Immunoprecipitation

HEK293T cells were cotransfected with the indicated plasmids as described in the figure legends. Total amounts of DNA were adjusted by adding an empty vector. At 48 h after transfection, cells were harvested and immunoprecipitation assay was performed as we reported previously (Lim et al., 2011).

### Immunoblot Analysis

Cells were harvested and lysed in buffer containing 50 mM Tris HCl (pH 7.5), 150 mM NaCl, 1% NP-40, 1 mM EDTA, 0.25% sodium deoxycholate, 1 mM Na_3_VO_4_, 1 mM sodium fluoride, 1 mM phenylmethylsulfonyl fluoride (PMSF), 1 mM β-glycerophosphate, and protease inhibitor cocktail (Roche) for 30 min on ice, and centrifuged at 12,000 rpm for 10 min at 4°C. The supernatant was collected and equal amounts of protein were subjected to SDS-PAGE and electrotransferred to a nitrocellulose membrane. The membrane was blocked in TBST buffer (20 mM Tris-HCl (pH 7.6), 150 mM NaCl, and 0.2% Tween 20) containing 5% non-fat dry milk for 1 h and then incubated overnight at 4°C with the indicated antibodies in TBST buffer containing 1% non-fat dry milk. The membrane was incubated with either horseradish peroxidase (HRP)-conjugated goat anti-rabbit antibody or goat anti-mouse antibody (Jackson ImmunoResearch Laboratories, West Grove, PA, United States) in TBST buffer for 1 h at room temperature. Proteins were detected using an ECL kit (Amersham Biosciences).

### Viral Infectivity Assay

Virus titers were calculated as focus-forming units (FFU) per milliliter as we described previously (Lim et al., 2011).

### Quantification of HCV RNA

Total RNAs were isolated from HCVcc-infected cells or cell culture media using RiboEx^TM^ LS reagent (Geneall Biotechnology) according to the manufacturer’s instructions. cDNAs were synthesized by using a cDNA synthesis kit (Toyobo). Quantitative real-time PCR (qRT-PCR) experiments were performed using an iQ5 multicolor real-time PCR detection system (Bio-Rad Laboratories, Hercules, CA, United States) as we reported previously (Lim et al., 2011). qRT-PCR primers of SPCS1 (P28972) and ApoE (P195285) were purchased from Bioneer (Korea). Expression level of RAD51 was measured using the primers: 5′– AGC GTT CAA CAC AGA CCA CCA G– 3′ (sense) and 5′–ATC AGC GAG TCG CAG AAG CAT C– 3′ (antisense).

### Nuclear and Cytoplasmic Fractionation

Huh7.5 cells were washed twice with cold PBS and then resuspended in buffer A (10 mM HEPES [pH 7.6], 10 mM KCl, 0.1 mM EDTA, 1 mM DTT, 0.5 mM PMSF). After incubation on ice for 15 min, the supernatant was collected by centrifugation at 16,000 × *g* for 5 min at 4°C and saved as cytoplasmic fraction. The pellet was solubilized in buffer B (20 mM HEPES [pH 7.6], 400 mM NaCl, 1 mM EDTA, 1 mM DTT, 1 mM PMSF). The dissolved pellet was further centrifuged at 16,000 × *g* for 5 min and then supernatant was collected and saved as nuclear fraction.

### Lipid Raft Isolation and Membrane Floatation Assay

Lipid raft isolation and membrane floatation assay were performed as described previously with a few modifications (Weaver et al., 2007). Briefly, HCV-infected cells were collected by scraping and then centrifuged for 5 min at 15,000 × *g*. The cells were resuspended in 1 ml of PBS and homogenized by 15 passages through a 25-gauge needle syringe. Protein concentrations were determined by the Bradford assay (Bio-Rad) according to manufacturer’s instructions. Equal amounts of proteins were centrifuged at 2,700 × *g* for 1 h. The remaining pellet was suspended in TNE buffer (25 mM Tris-HCl [pH 7.6], 150 mM NaCl, 5 mM EDTA) in the absence or presence of 1% Triton X-100 and rocked for 1 h on 4°C. The water-insoluble fraction was then centrifuged at 2,700 × *g* for 30 min. The pellet was resuspended in 0.5 ml of 40% OptiPrep solution (Sigma, 60% stock OptiPrep diluted in TNE) and placed in an ultracentrifuge tube (Hitachi). On the top of 40% layer, 3.5 ml of 30% OptiPrep solution was layered and then 0.5 ml of 5% OptiPrep solution was layered. The samples were centrifuged at 70,000 × *g* for 16 h at 4°C. Following centrifugation, 0.5 ml fractions were collected from the top to the bottom and each sample was numbered from 1 to 9. Equal amounts of protein from each fraction were loaded onto an 8–12% gradient SDS-PAGE and analyzed by immunoblot assay.

### Protease Protection Assay

A protease protection assay was performed as we reported previously (Saxena et al., 2012). Briefly, Huh7.5 cells were infected with Jc1. At 48 h post-infection, cells were harvested in ice-cold hypotonic buffer (10 mM Tris-HCl [pH 7.5] and 10 mM NaCl) and incubated for 10 min on ice. Cells were homogenized by 20 passages through a 25-gauge needle syringe. The cell lysates were centrifuged at 1,000 × *g* for 5 min at 4°C. The resulting post-nuclear supernatant (PNS) was incubated at 4°C in the absence or presence of 1% Trition X-100 for 20 min. The samples were either left untreated or treated with 20 or 40 μg/ml of proteinase K for 10 min. Proteinase K digestion was terminated with the addition of 2 mM PMSF for 10 min on ice. Samples were further centrifuged at 10,000 × *g* and proteins in both pellet (P) and supernatant (S) were analyzed by immunoblot assay.

### Coimmunoprecipitation of Rad51 with NS3 or HCV RNA

RNA immunoprecipitation assays were performed as previously reported (Dansako et al., 2013). Briefly, Huh7.5 cells infected with Jc1 were harvested in hypotonic buffer and then subjected to five cycles of freezing and thawing. Cells were then homogenized by 20 passages through a 25-gauge needle syringe. The PNS was resuspended in lysis buffer (PBS containing 0.1% NP-40, 400 U/ml of RNase inhibitor and protease inhibitor cocktail) and then incubated on ice for 30 min. Cell lysates were centrifuged at 18,000 × *g* for 30 min and then supernatant was incubated overnight at 4°C with either anti-Rad51 or anti-NS3 antibodies. Protein complexes were further precipitated with 40 μl of protein A beads (Invitrogen) for 2 h at 4°C. The beads were subsequently washed three times in washing buffer. RNAs were extracted from the immunoprecipitated complex using Trizol (Invitrogen) and then analyzed for HCV RNA by qRT-PCR.

### Immunofluorescence Assay

Huh7.5 cells infected with Jc1 were fixed with 4% paraformaldehyde in PBS for 10 min. Cells were permeabilized with 0.1% Triton X-100 in PBS for 10 min at room temperature and treated with 1% bovine serum albumin (BSA) in PBS for 1 h. Cells were incubated with anti-NS3 antibody and anti-Rad51 antibody. After three washes in PBS, cells were incubated with either fluorescein isothiocyanate (FITC)-conjugated goat anti-mouse IgG or tetramethylrhodamine isothiocyanate (TRITC)-conjugated donkey anti-rabbit IgG for 1 h at room temperature. Cells were counterstained with 4′, 6-diamidino-2-phenylindole (DAPI) to label nuclei. Samples were analyzed using the Zeiss LSM 700 laser confocal microscopy system (Carl Zeiss, Inc., Thornwood, NY, United States).

### Statistical Analysis

Data are presented as mean ± standard deviations (SDs). Student’s *t* test was used for statistical analysis. The asterisks on the figures indicate significant differences as noted in the legends.

## Results

### Rad51 Is Required for HCV Propagation

**FIGURE 1:**
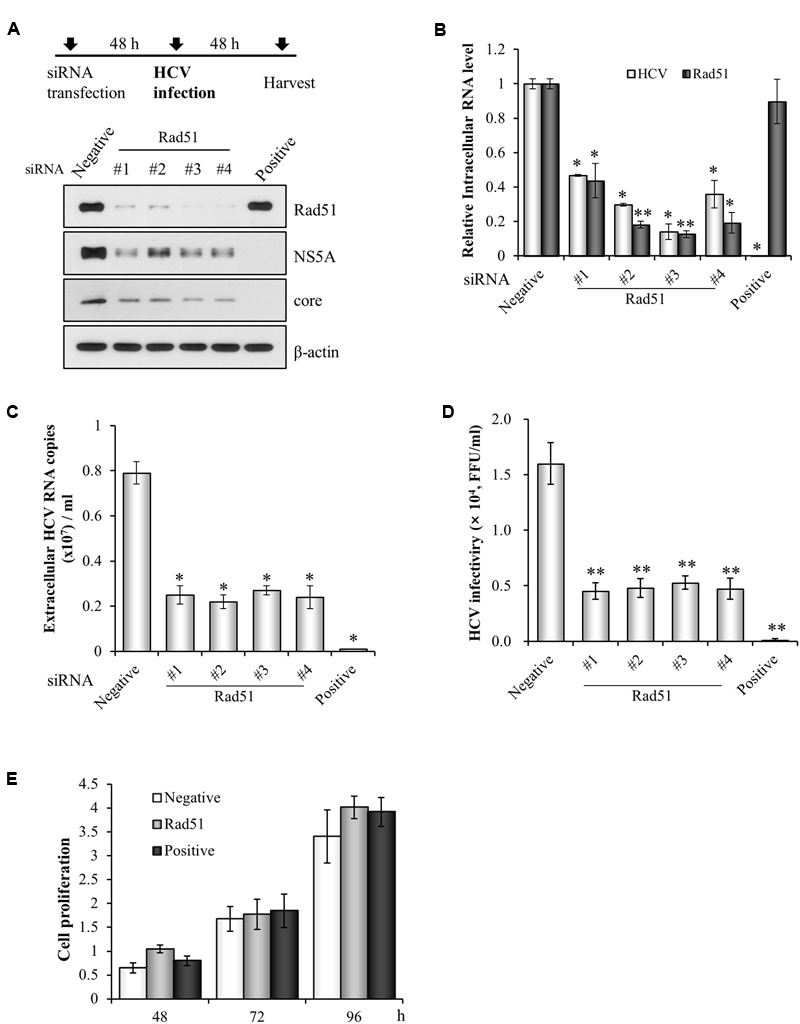
siRNA-mediated knockdown of Rad51 impairs HCV propagation. **(A)** Huh7.5 cells were transfected with 40 nM of the indicated siRNAs for 48 h and then infected with Jc1 at an MOI of 1 for 4 h. At 2 days post-infection, protein expression levels were analyzed by immunoblot analysis using the indicated antibodies. **(B,C)** Intracellular RNA levels of HCV and Rad51 **(B)** and extracellular HCV RNA levels **(C)** were determined by qRT-PCR. The asterisks indicate significant differences (^∗^*p* < 0.05, ^∗∗^*p* < 0.01) from the value for the negative control. Experiments were performed in triplicate. Error bars indicate standard deviations. **(D)** Naïve Huh7.5 cells were infected with cultured supernatant harvested from **(A)** for 4 h. At 48 h post-infection, extracellular HCV infectivity was determined by focus-forming assay. **(E)** Huh7.5 cells were transfected with the indicated siRNAs. Cell proliferation at the designated time points after transfection was determined by MTT assay. Data represent average from at least three independent experiments.

We previously screened a siRNA library targeting 131 genes that control cell cycle in HCVcc-infected cells and 13 pools were identified as candidate hits (Lim et al., 2011). Since Rad51 modulates HBV infection (Pasaje et al., 2011) and HIV integrase activity (Cosnefroy et al., 2012), we selected Rad51 to investigate its functional involvement in HCV propagation. For this purpose, Huh7.5 cells were transfected with siRNAs targeting Rad51 and then infected with Jc1. At 48 h post-infection, both protein and RNA levels were determined. Silencing of Rad51 attenuated HCV protein expression (**Figure 1A**) and significantly suppressed HCV RNA levels (**Figure 1B**). Moreover, silencing of Rad51 expression led to a strong inhibition of the extracellular HCV RNA levels (**Figure 1C**). To further investigate the effect of Rad51 on HCV infectivity, naïve Huh7.5 cells were infected with culture supernatant harvested from **Figure 1A**. **Figure 1D** showed that HCV infectivity was dramatically suppressed by knockdown of Rad51. We further demonstrated that treatment of the same concentration of siRNA displayed no cytotoxicity in HCV-infected cells, indicating that silencing effect was specific to Rad51 (**Figure 1E**). Collectively, these data suggest that Rad51 may be required for HCV propagation.

### Rad51 Is Involved in Later Stage of the HCV Life Cycle

**FIGURE 2:**
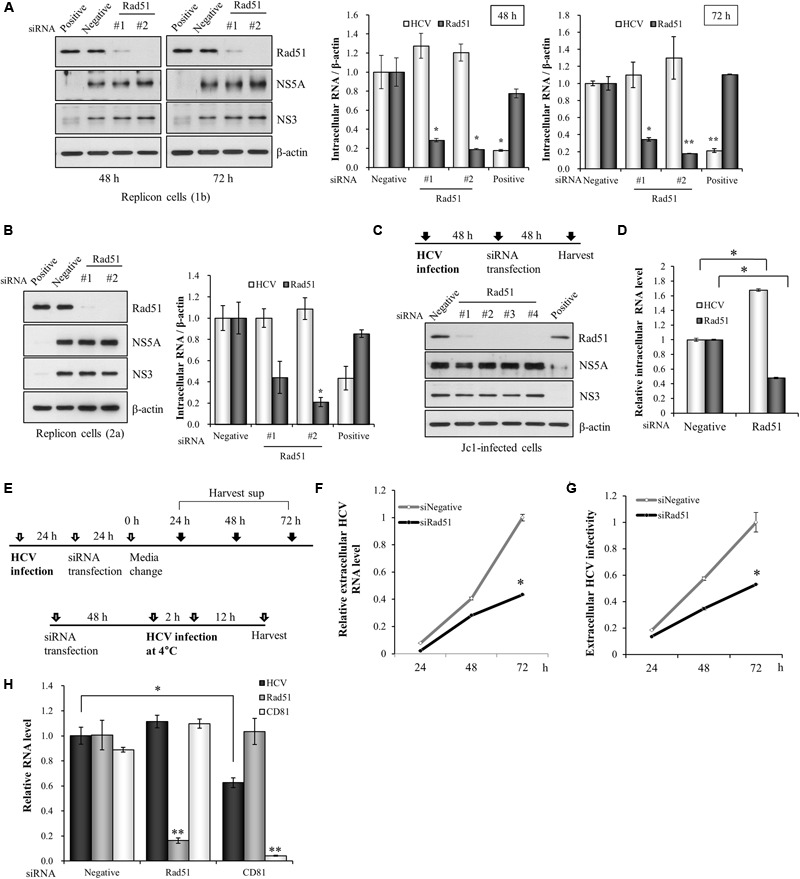
Rad51 is not required for HCV replication. **(A)** Huh7 cells harboring HCV subgenomic replicon derived from genotype 1b were transfected with the indicated siRNAs. At 48 h or 72 h after transfection, total cell lysates were immunoblotted with the indicated antibodies. HCV RNA and Rad51 mRNA levels were analyzed by qRT-PCR. **(B)** Huh6 cells harboring HCV subgenomic replicon derived from genotype 2a were transfected with the indicated siRNAs. At 72 h after transfection, total cell lysates were immunoblotted with the indicated antibodies. HCV RNA and Rad51 mRNA levels were analyzed by qRT-PCR. **(C,D)** Huh7.5 cells were infected with Jc1 (MOI = 1) for 4 h. At 48 h post-infection, cells were transfected with the indicated siRNAs. At 48 h after transfection, protein expression levels were determined by immunoblot analysis using the indicated antibodies **(C)** and intracellular RNA levels were analyzed by qRT-PCR **(D)**. Intracellular RNA levels were normalized to levels of β-actin mRNA. **(E)** Schematic illustration of the experimental design. **(F)** Huh7.5 cells infected with Jc1 (MOI = 1) were transfected with the indicated siRNAs for 24 h and then supplied with fresh media. The culture supernatants were harvested at the indicated time points and extracellular HCV RNA levels were analyzed by qRT-PCR. **(G)** Supernatants harvested at the various time points were used to infect naïve Huh7.5 cells and viral infectivity was determined by qRT-PCR. The asterisk indicates significant difference (^∗^*p* < 0.05) from the value for the negative siRNA control. Experiments were carried out in triplicate. Error bars indicate standard deviations. **(H)** Huh7.5 cells were transfected with the negative siRNA, Rad51-specific siRNA, and CD81-specific siRNA as a positive control, and then infected with HCV. At 12 h post-infection, HCV RNA levels were quantified by qRT-PCR. The asterisks indicate significant differences (^∗^*p* < 0.05, ^∗∗^*p* < 0.01) from the value for the negative siRNA control. Error bars indicate standard deviations.

To determine which step of HCV life cycle required Rad51, either Huh7 cells harboring HCV subgenomic replicon derived from genotype 1b (**Figure 2A**) or Huh6 cells harboring HCV subgenomic replicon derived from genotype 2a (**Figure 2B**) were transfected with the indicated siRNAs. At 48 h or 72 h after transfection, total cell lysates were immunoblotted with the indicated antibodies and HCV RNA levels were measured by qRT-PCR. As shown in **Figures 2A,B**, both viral protein expression levels and viral RNA levels in HCV replicons were not altered by knockdown of Rad51. To further verify this result, Huh7.5 cells were infected with Jc1 for 48 h and then transfected with the indicated siRNAs. At 2 days after transfection, total cell lysates were immunoblotted with the indicated antibodies. **Figure 2C** also showed that knockdown of Rad51 displayed no effect on HCV protein expression in HCV-infected cells. It was noteworthy that silencing of Rad51 increased intracellular HCV RNA level (**Figure 2D**). To further investigate the effect of Rad51 silencing on HCV propagation, we analyzed extracellular HCV RNA levels as depicted in **Figure 2E**. Huh7.5 cells infected with Jc1 were transfected with siRNAs and then extracellular HCV RNA levels in culture supernatants were determined at the indicated time points. As shown in **Figure 2F**, knockdown of Rad51 significantly reduced extracellular HCV RNA levels. We further demonstrated that viral infectivity was also significantly decreased by knockdown of Rad51 (**Figure 2G**). To investigate whether Rad51 was involved in entry step of the HCV life cycle, Huh7.5 cells were transfected with the negative siRNA, Rad51-specific siRNA, and CD81-specific siRNA as a positive control, and then infected with HCV. At 12 h post-infection, HCV RNA levels were quantified by qRT-PCR. As shown in **Figure 2H**, HCV RNA level was significantly decreased in CD81 knockdown cells. However, knockdown of Rad51 displayed no effect on HCV RNA level as compared with negative control, suggesting that Rad51 was not involved in early stage of HCV infection, including HCV entry step. These data suggest that Rad51 may be required for the assembly/release step but not replication step of the HCV life cycle.

### Rad51 Is Required for Viral Release but not Assembly of HCV

**FIGURE 3:**
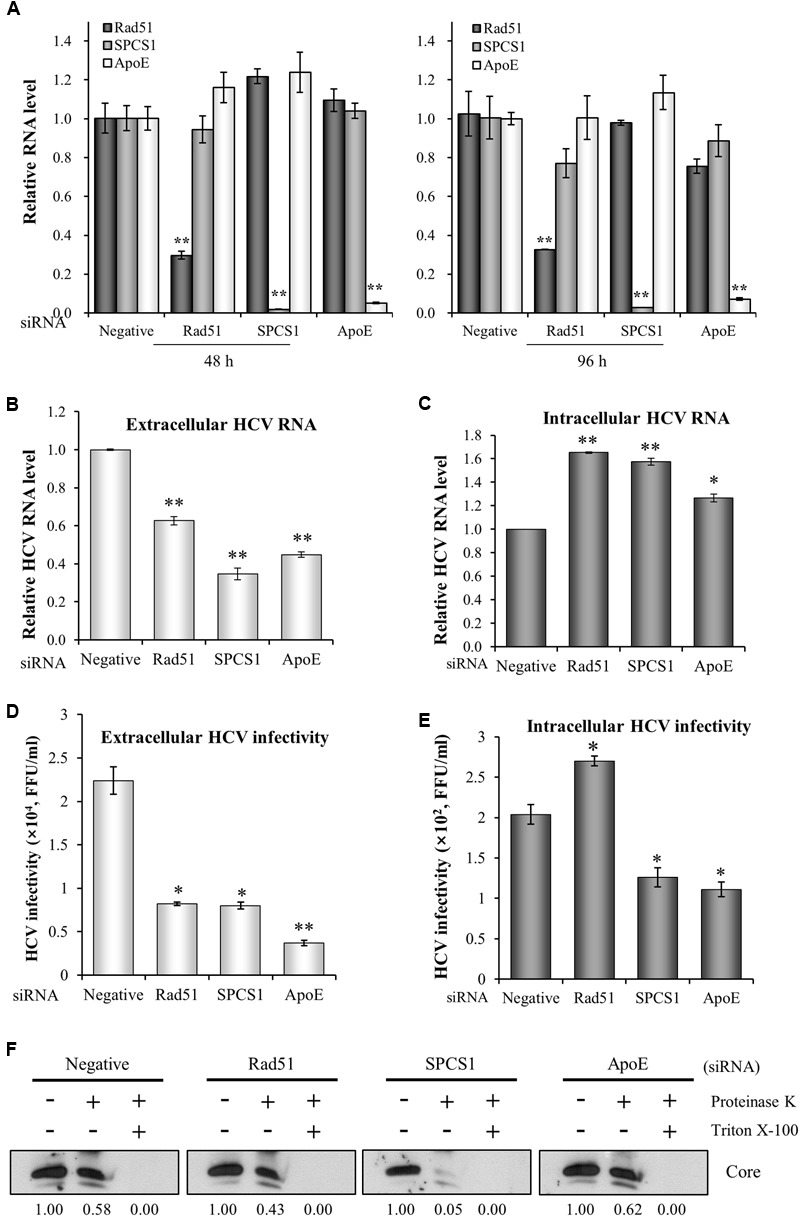
Rad51 is required for HCV release but not for HCV assembly. **(A)** Huh7.5 cells were transfected with the indicated siRNAs. At 48 h or 96 h after transfection, total RNAs were extracted and mRNA levels were analyzed by qRT-PCR. Huh7.5 cells were infected with Jc1 (MOI = 1) and then transfected with the indicated siRNAs. At 72 h after transfection, either extracellular HCV RNA level from the culture media **(B)** or intracellular HCV RNA level **(C)** was analyzed by qRT-PCR. **(D)** Naïve Huh7.5 cells were infected with HCV harvested from the supernatants **(B)**. Extracellular HCV infectivity was determined by focus-forming assay. **(E)** Naïve Huh7.5 cells were infected with intracellular HCV harvested from **(C)**. Intracellular HCV infectivity was determined by focus-forming assay. The asterisks indicate significant differences (^∗^*p* < 0.05, ^∗∗^*p* < 0.01) from the value for the negative siRNA control. Error bars indicate standard deviations. **(F)** Huh7.5 cells were infected with Jc1 and then transfected with the indicated siRNAs. At 48 h after transfection, cells were either left untreated or treated with proteinase K in the absence or presence of Triton X-100. The core protein levels were determined by immunoblot analysis using a rabbit anti-HCV core antibody. Both SPCS1 and ApoE were used as positive controls.

To precisely dissect the role of Rad51 in assembly/release of the HCV life cycle, Huh7.5 cells infected with Jc1 were transfected with siRNAs and then both extracellular and intracellular HCV RNA levels were analyzed by qRT-PCR. Since ApoE participates in both assembly and release steps of the HCV life cycle (Benga et al., 2010), ApoE was used as a positive control for assembly and secretion. It has been also reported that signal peptidase complex subunit 1 (SPCS1) is involved in assembly but not release of infectious virion (Suzuki et al., 2013) and thus SPCS1 was used as a control for assembly only. Huh7.5 cells were transfected with Rad51, SPCS1, or ApoE siRNAs. These siRNAs efficiently suppressed mRNA expression levels in cells harvested at 48 h or 96 h post-transfection (**Figure 3A**). As shown in **Figure 3B**, extracellular HCV RNA level was significantly decreased by knockdown of Rad51. We also showed that extracellular HCV RNA levels were substantially decreased by knockdown of either SPCS1 or ApoE. We next analyzed the intracellular HCV RNA levels. **Figure 3C** showed that intracellular HCV RNA levels were significantly increased in Rad51-, SPCS1-, and ApoE-enervated cells (**Figure 3C**). On the other hand, HCV infectivity was significantly decreased by knockdown of Rad51, SPCS1, and ApoE (**Figure 3D**). Of note, intracellular HCV infectivity was significantly increased in Rad51 knockdown cells, whereas it was significantly decreased in both SPCS1 and ApoE knockdown cells (**Figure 3E**). To further verify the functional involvement of Rad51 in HCV assembly, Huh7.5 cells infected with Jc1 were transfected with the indicated siRNAs and then intracellular core protein levels after proteinase K treatment were assessed by immunoblot assay. As shown in **Figure 3F**, core protein was protected from digestion by proteinase K in negative siRNA-treated cells. Likewise, core protein was highly resistant to proteinase K digestion in both Rad51 and ApoE knockdown cells. However, substantial amount of core protein was digested by proteinase K in SPCS1 knockdown cells, confirming that Rad51 was required for HCV release but not assembly of infectious HCV particles. All these data indicate that Rad51 is involved in release step of the HCV life cycle.

### Rad51 Interacts with both NS3 Protein and HCV RNA

**FIGURE 4:**
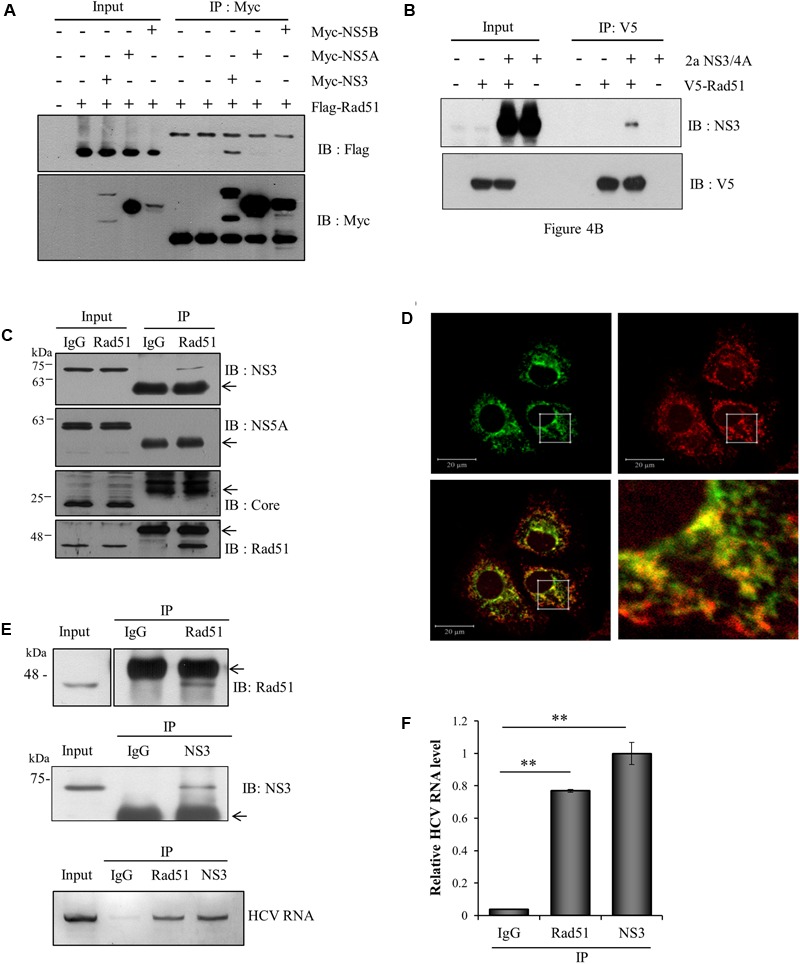
Rad51 interacts with both NS3 protein and HCV RNA. **(A)** HEK293T cells were cotransfected with each of Myc-tagged genotype 1b HCV protein expressing plasmid and Flag-tagged Rad51 as indicated. At 48 h after transfection, total cell lysates were immunoprecipitated with an anti-Myc antibody and then bound proteins were detected by immunoblot analysis using an anti-Flag antibody. Protein expressions of Myc-tagged NS3, NS5A, and NS5B in the same cell lysates were verified by immunoblot analysis using an anti-Myc antibody. **(B)** HEK293T cells were cotransfected with V5-tagged Rad51 and NS3/4A (genotype 2a) expressing plasmid. At 48 h after transfection, total cell lysates were immunoprecipitated with an anti-V5 antibody, and bound proteins were detected by immunoblot analysis using an anti-NS3 antibody. **(C)** Huh7.5 cells were infected with Jc1 (MOI = 1) for 4 h. At 72 h post-infection, total cell lysates were immunoprecipitated with either control IgG or an anti-Rad51 antibody and then bound proteins were detected by immunoblot analysis using the indicated antibodies. Arrows denote IgG. **(D)** Huh7.5 cells infected with Jc1 were fixed in 4% paraformaldehyde. Immunofluorescence assay was performed by using an anti-Rad51 polyclonal antibody and TRITC-conjugated donkey anti-mouse IgG to detect endogenous Rad51 (red); a rabbit anti-NS3 antibody and fluorescein isothiocyanate-conjugated goat anti-rabbit IgG were used to detect NS3 (green). Dual staining showed colocalization of endogenous Rad51 and NS5A as yellow fluorescence in the crop image. **(E)** (Upper) Huh7.5 cells were infected with Jc1. At 72 h post-infection, post-nuclear supernatants were prepared by centrifugation at 10,000 × *g*. The sample was lysed and immunoprecipitated with control IgG, anti-Rad51 antibody, and anti-NS3 antibody, respectively. Arrows indicate IgG. (Lower) HCV RNAs extracted from the protein-RNA complex were analyzed by qRT-PCR using HCV-specific primers. NS3 was used as a positive control. **(F)** HCV RNA levels in protein-RNA complex were quantified by qRT-PCR. The asterisks indicate significant differences (^∗∗^*p* < 0.01) from the value for the IgG control. Experiments were carried out in triplicate. Error bars indicate standard deviations.

To investigate how Rad51 was involved in HCV propagation, we first explored the possible interaction between Rad51 and viral proteins. HEK293T cells were cotransfected with Flag-tagged Rad51 and each of Myc-tagged genotype 1b HCV protein-expressing plasmid. Protein interaction was analyzed by coimmunoprecipitation assay. As shown in **Figure 4A**, Rad51 specifically interacted with NS3 protein. Since NS4A is required for enzymatic function of NS3, we wondered whether Rad51 could interact with enzymatically active NS3/4A (genotype 2a). For this purpose, we first verified the enzyme activity of NS3/4A by using Flag-tagged MAVS as a substrate (data not shown). Next, HEK293T cells were cotransfected with V5-tagged Rad51 and NS3/4A expressing plasmid. We confirmed that Rad51 bound to NS3/4A (**Figure 4B**). To further demonstrate protein interaction between Rad51 and viral proteins in the context of HCV replication, we performed immunoprecipitation assay using HCV-infected cells. **Figure 4C** showed that endogenous Rad51 specifically interacted with NS3 protein but not with other viral proteins in HCV-infected cells. In addition, endogenous Rad51 was colocalized with NS3 in HCV-infected cells (**Figure 4D**). Since Rad51 interacts with NS3 and NS3 is essential for viral RNA replication (Pang et al., 2002), we hypothesized that Rad51 might interact with HCV RNA in HCV replicating cells. To investigate this possibility, PNS prepared from Jc1-infected cells were immunoprecipitated with anti-Rad51 antibody and then HCV RNA coprecipitated with Rad51 protein was analyzed by qRT-PCR. As shown in **Figure 4E**, HCV RNA was readily detected in Rad51 precipitate prepared from Jc1-infected cells. Quantitative analysis data showed that ∼70% HCV RNA was coprecipitated with Rad51 as compared with NS3 which a positive control (**Figure 4F**). These data suggest that Rad51 may interact with HCV RNA though NS3 in HCV replicating cells.

### Rad51 Is Associated with NS3 on the Lipid Rafts in HCV Replicating Cells

**FIGURE 5:**
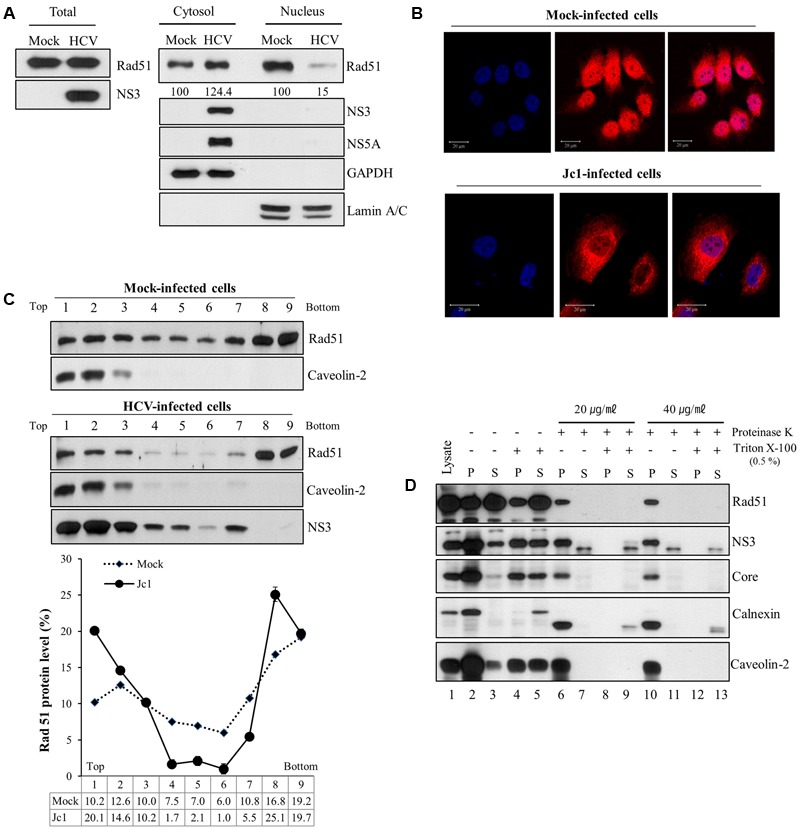
Rad51 is associated with lipid raft. **(A)** Huh7.5 cells were either mock infected or infected with Jc1 (MOI = 1) for 4 h. At 72 h post-infection, both cytosolic and nuclear fractions were prepared and then protein expression levels were determined by immunoblot analysis with the indicated antibodies. **(B)** Huh7.5 cells were either mock-infected or infected with Jc1 for 4 h. At 2 days post-infection, cells were fixed with 4% paraformaldehyde, and immunofluorescence staining was performed by using an anti-Rad51 antibody and TRITC-conjugated donkey anti-rabbit IgG to detect Rad51 (red). Cells were counterstained with DAPI to label nuclei (blue). **(C)** Huh7.5 cells were either mock- or infected with Jc1 for 4 h. At 72 h post-infection, cells were homogenized and then centrifuged to remove supernatants. The pellets were subjected to membrane floatation centrifugation. (Top panels) Nine fractions were collected and protein levels in each fraction were determined by immunoblot analysis with the indicated antibodies. (Bottom panel) The band intensities of Rad51 protein in each fraction were quantified using Image J software. **(D)** Protease protection assay. Post-nuclear supernatants prepared from Huh7.5 cells infected with Jc1 were either left untreated (lanes 2–3), treated with 0.5% Triton X-100 (lanes 4–5), proteinase K (lanes 6–7 and 10–11), or co-treated with Triton X-100 and proteinase K (lanes 8–9 and 12–13). The samples were further centrifuged at 10,000 × *g* and both pellets (P) and supernatants (S) were determined by immunoblot analysis with the indicated antibodies. Lane 1, total cell lysate.

Next, we examined whether subcellular localization of Rad51 was altered by HCV infection. For this purpose, both cytosolic and nuclear fractions prepared from either mock- or Jc1-infected cells were immunoblotted with an anti-Rad51 antibody. Rad51 level in nuclear fraction was dramatically decreased, whereas Rad51 level in cytosolic fraction was increased in Jc1-infected cells as compared with mock-infected cells (**Figure 5A**). To further confirm the alteration of subcellular distribution of Rad51 in HCV-infected cells, we performed immunofluorescence assay using an anti-Rad51 antibody. As shown in **Figure 5B**, Rad51 proteins were mainly localized in nuclei of mock-infected cells. Notably, Rad51 protein levels were reduced in the nuclei and accumulated in the cytoplasm of Jc1-infected cells. These results suggest that HCV infection may modulate subcellular localization of Rad51 to facilitate viral propagation. To investigate whether Rad51 was co-fractionated with NS3 in HCV replicating cells, we performed a membrane flotation assay. As expected, majority of caveolin-2, a lipid raft marker, was localized at fractions 1 to 3 (**Figure 5C**, top panels). We showed that 44.9% of the total amount of Rad51 protein was co-fractionated with NS3 protein on the lipid rafts in the HCV-infected cells (**Figure 5C**, bottom panel). However, 32.8% of the total amount of Rad51 protein was co-fractionated on the lipid rafts in the mock-infected cells. Since HCV RNA replication takes place on the lipid rafts, we further investigated whether Rad51 was localized on the lipid rafts by protease protection assay. PNSs were prepared from Jc1-infected cells and then treated with either Triton X-100 or proteinase K alone, or co-treated with Triton X-100 and proteinase K. **Figure 5D** showed that calnexin, an ER marker, was readily degraded by proteinase K (lane 6), whereas Rad51, NS3, core, and caveolin-2 were highly resistant to proteinase K (lanes 6 and 10) in Jc1-infected cells. These data indicate that Rad51 is associated with NS3 on the lipid rafts in HCV-infected cells.

## Discussion

Hepatitis C virus infection is characterized by a high propensity to chronicity. Although the molecular mechanisms of chronicity remain poorly understood, chronic infection with HCV often leads to liver cirrhosis and HCC. The HCV life cycle is highly dependent on host cell factors. Since the mutation rates in viral genomes are considerably higher than those in cellular genes, targeting of host factors may provide an advantage for the development of drugs with a high barrier to resistance. To identify the cellular factors involved in HCV replication, we previously screened a siRNA library targeting the cell cycle in HCVcc-infected cells (Lim et al., 2011). Among the candidate hits that decreased extracellular HCV RNA levels, we selected and characterized the gene encoding Rad51.

Rad51 is the main element involved in the homologous recombination process (Sung et al., 2003). Rad51 catalyzes the homology search and the strand exchange with a homologous sequence and thus ensures the accurate repair of the DSB (Haaf et al., 1995). Rad51 has been involved in many viral infections. Rad51 interacts with Begomovirus replication initiator protein to improve viral replication (Suyal et al., 2013). In astrocytes, increased levels of Rad51 led to stimulation of HIV-1 LTR activity through collaboration with HIV-1 Tat, the transcription factors C/EBPβ, and CHOP (Chipitsyna et al., 2006). In the present study, we showed that knockdown of Rad51 prior to HCV infection significantly reduced HCV protein expression as well as intracellular and extracellular HCV RNA levels. However, silencing of Rad51 after virus infection displayed no effect on HCV protein expression levels. Although extracellular HCV RNA levels were significantly decreased, intracellular HCV RNA levels were increased by knockdown of Rad51 in HCV-infected cells. These data suggest that Rad51 may be involved in late step of the HCV life cycle. In addition, we showed that HCV core was substantially protected from proteinase K digestion in both Rad51- and ApoE-knockdown cells but not in SPCS1-enervated cells, indicating that Rad51 was required for viral release but not assembly of HCV particles.

Next, we investigated whether Rad51 could interact with HCV proteins. Protein interaction between endogenous Rad51 and HCV NS3 protein was confirmed by coimmunoprecipitation assays. We therefore tempted to speculate that HCV may exploit Rad51 via NS3 to regulate infectious viral particles. It has been also reported that Rad51 is recruited onto several kinds of nucleotides to form the nucleofilament (Baumann and West, 1998). We showed that more than 70% of HCV RNA was co-precipitated with Rad51 as compared with NS3 as a positive control. After DSB, Rad51 protein is loaded onto the ssDNA to form a contiguous nucleoprotein filament (Baumann and West, 1999). We therefore asked whether HCV infection altered the subcellular localization of Rad51. We showed that HCV infection interrupted the translocation of Rad51 from cytoplasm to nucleus and recruited Rad51 to the lipid rafts. In fact, Rad51 interacted with both NS3 and HCV RNA. These findings led us to examine whether Rad51 was associated with HCV replication complex on the lipid rafts. By employing both protease protection and membrane flotation assays, we demonstrated that Rad51 was protected from protease degradation and co-fractionated with HCV proteins. These data suggest that Rad51 may be recruited to the HCV replication complex through interaction with both HCV RNA and NS3. Recently, several groups reported that NS3 regulates the production of infectious HCV particles. McGivern et al. (2015) reported that NS3 regulates virus assembly and maturation of HCV. NS3 is also involved in HCV infectivity via linker region between protease and helicase domain since linker region is implicated in interaction with other viral and host proteins (Kohlway et al., 2014). Moreover, the late step of the HCV life cycle is impaired in certain drug-resistant mutants of protease domain of NS3 (Shimakami et al., 2010). These studies suggest that NS3 may interact with cellular proteins to regulate late stage of the HCV life cycle. Since Rad51 interacts with NS3 and NS3 is involved in the production of infectious HCV particles, knockdown of Rad51 could impair the production of infectious HCV particles. In this aspect, protein interaction between NS3 and Rad51 is critical for HCV release. Collectively, HCV may coopt host Rad51 protein through NS3 to facilitate the release of infectious particles and thus Rad51 may be a potential therapeutic target for HCV.

## Author Contributions

All authors have given approval to the final version of the manuscript. KS performed experiments, analyzed data, and wrote the manuscript. LP, TN, J-WC, and Y-SL performed experiments. KS and TL analyzed data. Y-SL and SH designed experiments. SH supervised the study and wrote the manuscript.

## Conflict of Interest Statement

The authors declare that the research was conducted in the absence of any commercial or financial relationships that could be construed as a potential conflict of interest.

## Abbreviations

HCC: hepatocellular carcinoma
HCV: hepatitis C virus
HCVcc: cell culture grown HCV
